# Research on Orbital Angular Momentum Recognition Technology Based on a Convolutional Neural Network

**DOI:** 10.3390/s23020971

**Published:** 2023-01-14

**Authors:** Xiaoji Li, Leiming Sun, Jiemei Huang, Fanze Zeng

**Affiliations:** Key Laboratory of Cognitive Radio and Information Processing, Ministry of Education, Guilin University of Electronic Technology, Guilin 541004, China

**Keywords:** orbital angular momentum, ocean turbulence, convolutional neural networks

## Abstract

In underwater wireless optical communication (UWOC), a vortex beam carrying orbital angular momentum has a spatial spiral phase distribution, which provides spatial freedom for UWOC and, as a new information modulation dimension resource, it can greatly improve channel capacity and spectral efficiency. In a case of the disturbance of a vortex beam by ocean turbulence, where a Laguerre–Gaussian (*LG*) beam carrying orbital angular momentum (OAM) is damaged by turbulence and distortion, which affects OAM pattern recognition, and the phase feature of the phase map not only has spiral wavefront but also phase singularity feature, the convolutional neural network (CNN) model can effectively extract the information of the distorted OAM phase map to realize the recognition of dual-mode OAM and single-mode OAM. The phase map of the Laguerre–Gaussian beam passing through ocean turbulence was used as a dataset to simulate and analyze the OAM recognition effect during turbulence caused by different temperature ratios and salinity. The results showed that, during strong turbulence $C_{n}^{2}=1.0\times 10^{-13}K^{2}m^{-2/3}$, when different ω = −1.75, the recognition rate of dual-mode OAM (ℓ = ±1~±5, ±1~±6, ±1~±7, ±1~±8, ±1~±9, ±1~±10) had higher recognition rates of 100%, 100%, 100%, 100%, 98.89%, and 98.67% and single-mode OAM (ℓ = 1~5, 1~6, 1~7, 1~8, 1~9, 1~10) had higher recognition rates of 93.33%, 92.77%, 92.33%, 90%, 87.78%, and 84%, respectively. With the increase in ω, the recognition accuracy of the CNN model will gradually decrease, and in a fixed case, the dual-mode OAM has stronger anti-interference ability than single-mode OAM. These results may provide a reference for optical communication technologies that implement high-capacity OAM.

## 1. Introduction

The first section mainly introduces the background and research significance of orbital angular momentum (OAM) optical communication and investigates the research status of convolutional neural network (CNN) recognition of OAM.

With the rapid development of underwater optical communication technology, the vortex beam carrying OAM is a new type of beam, and the topological charge can theoretically be any integer, so without increasing the spectral bandwidth, the information transmission rate and channel capacity of the system [1,2,3] can be greatly improved by the OAM multiplexing mode, which can effectively solve the problems of low information transmission rate and insufficient bandwidth common in underwater communication and has great potential and wide application prospects. The most representative OAM beam is the Laguerre–Gaussian (*LG*) beam, and the different OAM modes are orthogonal to each other. This indicates that different OAM modes do not interfere with each other during transmission, so OAM light can be applied to the compilation code and the multiplexing transmission of wireless optical communication [4,5,6,7] to meet the growing demand for information transmission capacity; in fact, the information carried by OAM is limited, which is related to the OAM beam’s own anti-interference ability and transmission interference, facing the complex and changeable underwater environment, *LG* crosstalk occurs between the orbital angular momentum of the beam during transmission, which makes it more difficult for the receiver to identify OAM, so OAM identification is extremely critical to the communication system.

A variety of detection methods have been proposed to identify OAM patterns, mainly including interferometer diffraction methods [8,9,10], which can identify OAM patterns by observing the interference fringe distribution. The diffraction method detects OAM patterns by designing special diffractive optics and measuring the far-field diffraction pattern after the vortex beam passes through the diffractive element. In addition, the support vector machine learning model can achieve the recognition effect by extracting sample features [11,12,13,14], but when the sample size is large, the recognition effect is saturated. The spiral wavefront phase of the vortex beam is susceptible to turbulence, resulting in pattern dispersion and intensity distortion, which in turn leads to the distortion of interference or diffraction fringes. With the increase in turbulence intensity and transmission distance, the distortion of the interference of light intensity distribution becomes more and more serious [15,16,17,18]. This increases the difficulty of OAM pattern recognition. In order to further expand the research and application scope of the vortex beam, it is necessary to seek an effective method that can quickly detect large-scale OAM patterns with high accuracy and strong turbulence resistance. CNN has more advantages in image processing due to its special convolutional structure and its powerful information extraction ability and has been widely used in OAM pattern recognition. A previous study [19], proposed and examined an OAM pattern recognition method based on a convolutional neural network, constructed an eight-layer CNN with a complex feature extraction ability, and trained it by setting Gaussian beams to interfere with the intensity mode of vortex beams, giving it strong resistance to turbulence. After supervised training on large sample sets, the CNN model demonstrated an excellent network generalization ability and was able to detect the mode range well [−50,50]. The simulation results show that, under the influence of weak turbulence and medium turbulence, the average recognition rate exceeds 99%. Even during strong turbulence, the accuracy reaches 98.54%. Another study [20], deepened the network and used the residual learning framework to address degradation. By testing the demodulation performance of OAM systems with 4-ary, 8-ary, 10-ary, and 16-ary, the generalization ability of training models using different training sets to adapt to unknown turbulence environments was analyzed. Numerical simulations show that at the level of strong turbulence at 2000 m free space, the demodulation accuracy of 4-ary, 8-ary, 10-ary, and 16-ary OAM systems is 100.0%, 99.5%, 99.2%, and 99.0%, respectively. A further study [21], utilized a 6-layer CNN for OAM identification during atmospheric turbulence in order to effectively realize the recognition of OAM patterns by feature extraction of the intensity distribution of the received *LG* beam. We examine our designed CNNs against different turbulence levels, transmission distances, and pattern intervals and attempted to compare their performance in identifying individual OAM patterns and multiplexing OAM patterns. The coaxial multiplexed OAM mode is able to obtain high recognition accuracy even under the long transmission distance of strong turbulence, i.e., about 96.25%.

At present, the use of CNN for OAM recognition is based on the light intensity map as the training object, which can identify OAM patterns well [22,23,24,25,26,27]; however, the beam light intensity is easily destroyed by turbulence and dispersed. The phase characteristics of the phase map not only have a spiral wavefront but also phase singularity features, and as more features can better resist the interference of turbulence, this paper proposes the selection of received LG. The phase map of the beam is extracted for features, and the convolutional neural network model is based on the convolutional neural network model in order to realize the identification of OAM during ocean turbulence. The results show that the acquisition of phase map features can realize OAM pattern recognition, which provides a reference for OAM pattern recognition.

## 2. Materials and Methods

Section 2 introduces the formula of the *LG* beam and the definition of the single mode and dual mode of *LG* beam. Along with the basic principle of the ocean turbulence random phase screen, the ocean turbulence channel model was constructed, and the phase distribution characteristics of *LG* beam single mode and dual mode were analyzed.

### 2.1. LG Beam

In column coordinates, the expression of the light field propagating by the *LG* beam along the z-axis can be expressed as [21]:

(1) $$\begin{array}{l} LG_{p}^{\ell }(r,\theta ,z)=\sqrt{\frac{2p!}{\left(\pi (p+|\ell |)!\right)}}{\left[\frac{r\sqrt{2}}{w(z)}\right]}^{\left|\ell \right|}L_{p}^{\ell }\left[\frac{2r^{2}}{w^{2}(z)}\right]\exp\left[\frac{-r^{2}}{w^{2}(z)}-\frac{ikr^{2}z}{2R(z)}\right]\\ \;\;\;\;\;\;\;\;\;\;\;\;\;\;\;\;\;\;\;\;\times \exp\left[i(2p+\ell +1)\tan^{-1}\frac{z}{z_{R}}\right]\exp(i\ell \theta ) \end{array}$$

where $w(z)=w_{0}\sqrt{1+{\left(z/z_{R}\right)}^{2}}$ is the radius of the girdle after transmitting the distance of z; $z_{R}=kw_{0}^{2}/2$ indicates the Rayleigh length; $w_{0}$ is the zero-order girdle radius, that is, the girdle radius when the transmission distance z = 0; k=2π/λ denotes beam; ℓ is the topological charge value of the beam, which represents the phase change of the beam along the direction angle; p is the radial factor that represents the phase change that occurs in the beam along the radius; ${\left[r\sqrt{2}/w\left(z\right)\right]}^{\left|\ell \right|}$ represents a vortex core function affected by a phase singularity; $L_{p}^{\ell }$ denotes Laguerre polynomials; exp(iℓθ) spiral phase factor; *i* is the imaginary unit; and θ is the directional phase angle, which indicates that the beam carries orbital angular momentum.

There the dual-mode *LG* beam can be expressed as:

(2) $$LG^{\pm \ell }=LG^{-\ell }+LG^{+\ell }$$

where $LG^{-\ell }$ represents a single-mode *LG* beam with a reverse spiral, $LG^{+\ell }$ represents a single-mode *LG* beam with a forward spiral, and $LG^{\pm \ell }$ represents a dual-mode *LG* beam, for example, $LG^{-4}$ is an *LG* beam with ℓ= −4, $LG^{\pm 4}$ is an *LG* beam with ℓ= 4, and $LG^{\pm 4}$ is a dual-mode *LG* beam with ℓ= ±4.

### 2.2. Ocean Turbulence Random Phase Screen Model

The influence of ocean turbulence on beam transmission is simulated by passing the beam through a series of equally spaced random phase screens, and the random phase screen model of ocean turbulence is constructed by power spectrum inversion.

The common refractive index fluctuation spectrum of seawater was proposed by Nikishov [28] et al. using the expression:

(3) $$\begin{array}{l} \Phi (k_{x},k_{y})=0.388\times 10^{-8}C_{n}^{2}(\sqrt{k_{x}^{2}+k_{y}^{2}}{)}^{-11/3}[1+2.35(\sqrt{k_{x}^{2}+k_{y}^{2}}\eta )2/3]\times \\ \;\;\;\;\;\;\;\;\;\;\;\;\;\;\;\;(e^{-A_{T}\delta }+\omega^{-2}e^{-A_{S}\delta }-2\omega^{-1}e^{-A_{TS}\delta }) \end{array}$$

where $C_{n}^{2}=10^{-8}\chi_{T}\varepsilon^{-1/3}$ is the equivalent temperature structural parameter, ε is the kinetic energy dissipation rate per unit volume of seawater, and the value range is $\left[10^{-10}m^{2}/s^{3},10^{-1}m^{2}/s^{3}\right]$;$\chi_{T}$ is the mean square seawater temperature dissipation rate, and the value range is $\left[10^{-10}K^{2}/s,10^{-4}K^{2}/s\right]$; ω is the turbulence caused by the change in temperature gradient and salinity gradient, and the value range is [−5,0]; and η is the Kolmogorov microscale, the value range is $\left[6\times 10^{-3}m,0.01m\right]$, and regarding the depths of seawater, on the Kolmogorov scale, the size of η is close to 0.01 m. The other parameters of the equation are set to: $A_{T}=1.863\times 10^{-2}$, $A_{S}=1.9\times 10^{-4}$, $A_{TS}=9.41\times 10^{-3}$, $\delta =8.284\times {\left(\sqrt{k_{x}^{2}+k_{y}^{2}}\eta \right)}^{3/4}+12.978{\left(\sqrt{k_{x}^{2}+k_{y}^{2}}\eta \right)}^{2}$. 

Firstly, the method of generating the phase screen based on power spectrum inversion methods generates a zero mean, and a unit variance of 1 in the frequency domain Hermitian complex Gaussian random number matrix $H\left(k_{x},k_{y}\right)$ uses the phase spectrum of seawater that conforms to the Kolmogorov spectrum of ocean turbulence. The function filters $F_{\Phi }\left(k_{x},k_{y}\right)$ and $H\left(k_{x},k_{y}\right)$ perform the inverse Fourier transform to obtain the random phase screen of ocean turbulence ϕ(x,y), which can be expressed as:

(4) $$\phi \left(x,y\right)=C\sum_{k_{x}}\sum_{k_{y}}H\left(k_{x},k_{y}\right)\sqrt{F_{\Phi }\left(k_{x},k_{y}\right)}\exp\left[j\left(k_{x}x+k_{y}y\right)\right]$$

A matrix of Gaussian random numbers with a mean of 0 and variance N×N of 1 is generated by randn(), and then, a Fourier transform is performed $H\left(k_{x},k_{y}\right)$.

The seawater phase spectrum $F_{\Phi }\left(k_{x},k_{y}\right)$ on a sliced surface perpendicular to the propagation direction of the beam can be expressed as:

(5) $$F_{\Phi }\left(k_{x},k_{y}\right)=2\pi k^{2}\Delta z\Phi (k_{x},k_{y})$$

where is the Δz propagation distance of the beam and $\Phi \left(k_{x},k_{y}\right)$ is the refractive index fluctuation spectrum of seawater.

**Figure 1:**
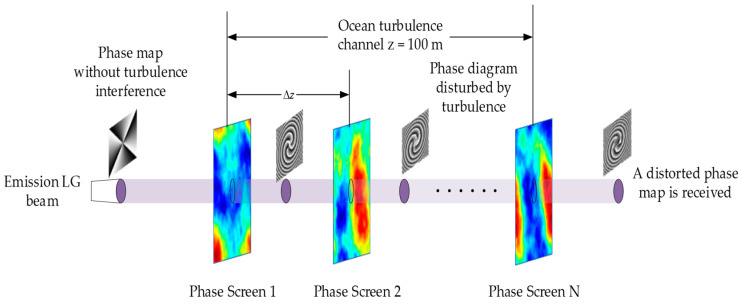
Random phase screen model of ocean turbulence.

The random phase screen model of ocean turbulence is shown in Figure 1, the *LG* beam is generated at the transmitting end, the *LG* beam passes through the equally spaced random phase screen, and the receiving end receives the distorted *LG* beam phase map.

Suppose the plane where the phase display is located is the XY plane and the beam is transmitted in the Z axial direction. In the spatial domain, the light field of the initial beam is $U_{0}(x,y)$. $U_{0}(x,y)$ is a complex number whose modulus magnitude indicates the intensity of the light field, and the angle represents the spatial phase of the light field. Assuming that the beam is transmitted in a free space channel, if the transfer function in the spatial frequency domain is $U_{prop}(k_{x},k_{y})$, the beam is transmitted only in free space until the first phase screen is reached. The light field when it reaches the first phase screen can be expressed as:

(6) $$U_{1-}(x,y)=F^{-1}\{F[U_{0}(x,y)]\times U_{prop}(k_{x},k_{y})\}$$

where $k_{x}$ and $k_{y}$ are the frequency components of the X axis and Y axis direction in the spatial frequency domain, F represents the Fourier transform, and $F^{-1}$ represents the inverse Fourier transform. $U_{prop}(k_{x},k_{y})$ is a free-space transfer function whose expression is as follows:

(7) $$U_{prop}=\exp(i\Delta z\sqrt{k^{2}-k_{x}{}^{2}-k_{y}{}^{2}})$$

After the beam passes through the phase screen, the spatial phase of its light field is affected by the phase screen model, and the light field changes:

(8) $$U_{1+}(x,y)=U_{1-}(x,y)\times i\varphi (x,y)$$

where φ(x,y) is the distribution function of the random phase screen.

**Figure 2:**
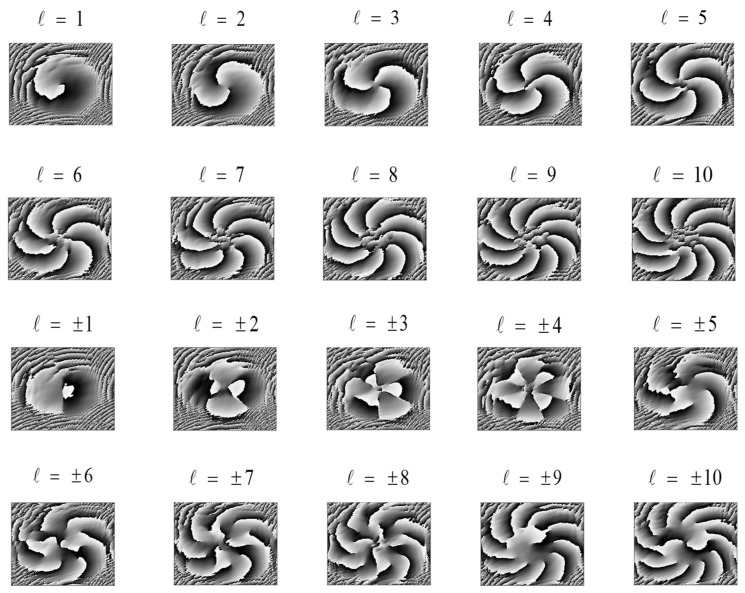
Phase distribution of *LG* beams with turbulent disturbances.

The phase distribution of an *LG* beam with turbulent disturbances is shown in Figure 2. As can be seen from the figure, in the absence of turbulence or after turbulence, the phase distribution is destroyed, and as the intensity of turbulence increases, its phase distribution distortion becomes more pronounced, which severely limits the effective recognition of OAM patterns.

## 3. Convolutional Neural Networks Recognize OAM

Section 3 mainly introduces the composition of the experimental CNN model, the feature extraction of the phase map, and the experimental analysis of OAM recognition.

### 3.1. Construction of Convolutional Neural Networks

CNN is a multilayer perceptron similar to artificial neural network, and the CNN model architecture includes the following: input layer, convolution layer, pooling layer, and fully connected layer. The input layer preprocesses the raw image data, deaveraging and normalizing the data. The function of the convolutional layer is to extract features and enhance the original signal features, and the purpose of the convolution kernel is to extract feature information from the phase map, play the role of feature extractor, and obtain multiple feature maps. The pooling layer performs advanced feature extraction on the feature images output by the convolutional layer, reducing the weight parameters required for network training. Pooling operations include the maximum pool and the average pool, where the largest pool takes the maximum value of the sampling point and the average pool takes the average. The fully connected layer is a linear transformation and nonlinear transformation of the features obtained by the convolutional layer and the pooling layer, and its functions are the classification layer and regression layer. In CNN model construction, activation functions are used to introduce nonlinear effects into the model, enabling the model to deal with complex problems. When the fully connected layer is the classification layer and the regression layer, the activation function can be the Softmax or the Relu function.

The mathematical expression for the Relu function is [21] as follows:

(9) f(x)=max(0,x)

In Formula (9), when x takes the value is (−∞, 0), the output value of the Relu function is 0, and when the x value is greater than 0, the output value of the Relu function is equal to the input value.

The mathematical expression of the Softmax function is as follows:

(10) $$\sigma {\left(z\right)}_{j}=\frac{e^{z_{j}}}{\sum_{k=1}^{K}e^{z_{k}}}$$

In Formula (10), the probability value of the j-th output is calculated, where j = 1,2,…,K, indicating that there are a total of K categories.

During training, the loss function is a criterion for assessing how well the model fits. To optimize the CNN training results, it is necessary to minimize the value of the loss function. Here, the cross-entropy function is used as the loss function to optimize the classification performance of the CNN model, and it can be expressed as:

(11) $$L(f(X,\theta ),Y)=-\sum y_{i}\ln f(x_{i},\theta )$$

**Figure 3:**
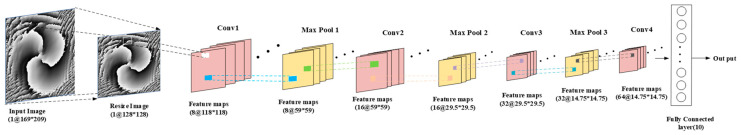
Structure diagram of CNN model recognition OM.

In the structural design of CNN, if the CNN model is too deep, the computational complexity will be large, which may produce serious overfitting, and if the CNN model is too shallow, it will not be able to effectively extract the features of the image, resulting in poor recognition accuracy. Therefore, the final network model is shown in Figure 3, as four convolutional layers, three maximum pooling layers, and one fully connected layer. In order to reduce the computational complexity of the network, the input layer normalizes the size of the input image to 128 × 128, and batches normalize it after each convolutional layer and use Relu as an excitation function to ensure that the value of the feature map is within a reasonable range. The convolutional layer output can be input to the fully connected layer as the different features of the input image, and the soft max classifier converts the feature map into the desired output to obtain the OAM mode information of the image.

### 3.2. Phase Map Feature Extraction

**Figure 4:**
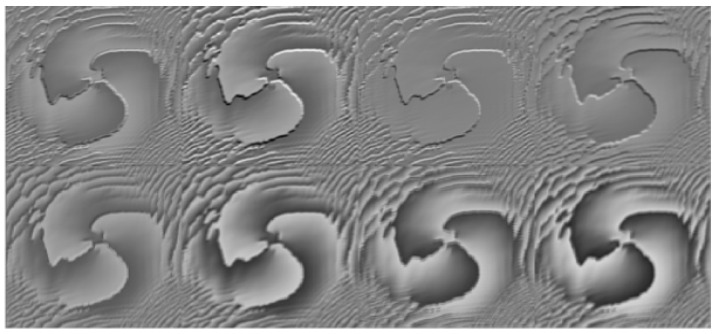
Feature diagram of the first convolutional layer.

**Figure 5:**
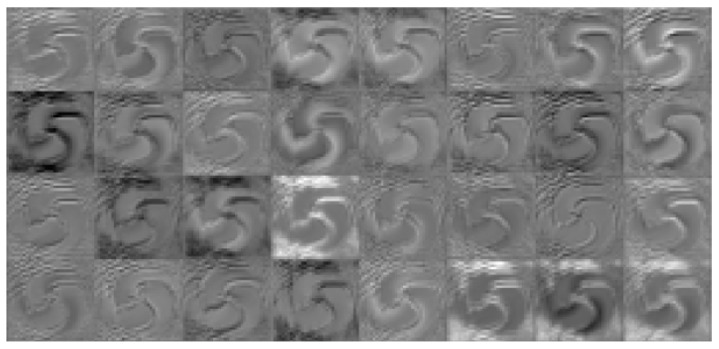
Feature map of the third convolutional layer.

The feature extraction aspect of the convolutional neural network is mainly carried out through the convolutional layer, which can extract different features from the image. With a deepening of the number of layers, low-level features are continuously fused to form high-level features, for example, the edge features extracted at the beginning can be fused to form high-level shape features, and through the deep-level learning network, the process can master enough feature information for judgment and can finally output reliable results. Taking the first convolutional layer and the third convolutional layer as examples, as shown in Figure 4, the convolutional layer has eight convolution kernels, the output feature map has eight channels, each channel can be regarded as a grayscale map, and the convolutional layer in Figure 5 has 32 feature maps.

There are eight feature maps in the above figure, and each feature map contains the low-level feature information extracted by the convolutional layer from the original image.

The above figure visualizes the feature maps of layer 1 and layer 3. In Figure 4, and it can be seen that convolutional layer 1 is visible. The extracted features are more specific and more in line with human vision. In Figure 5, the feature map of the third convolutional layer is highly abstracted, but the singularity region of the OAM beam phase map can be retained, which is also part of the efficiency of deep neural network classification recognition. The following is the interpretation of part of the feature map of convolutional layer 1.

**Figure 6:**
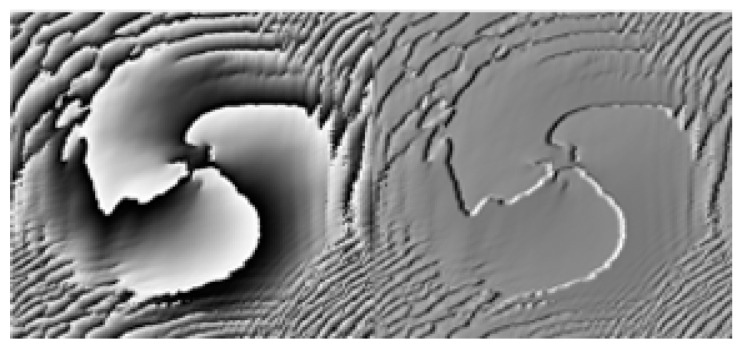
Feature map.

In Figure 6, The activation values on the four channels are extracted and resized to the dimensions of the original image. It can be seen that where the original image brightness transition contrast is obvious, there is a high-contrast arc at the corresponding position on the fourth channel. From this, it can be seen that channel 4 is “looking for” the characteristics of contrast.

**Figure 7:**
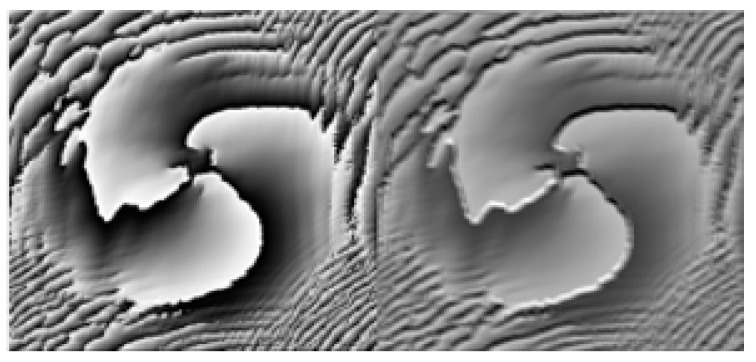
Feature map.

In Figure 7, the activation value on channel 1 is extracted, and the feature value area corresponding to the black area in the original figure is presented as black, which demonstrates that the first channel is “looking for” black features.

### 3.3. OAM Recognition Simulation Results and Analysis

**Figure 8:**
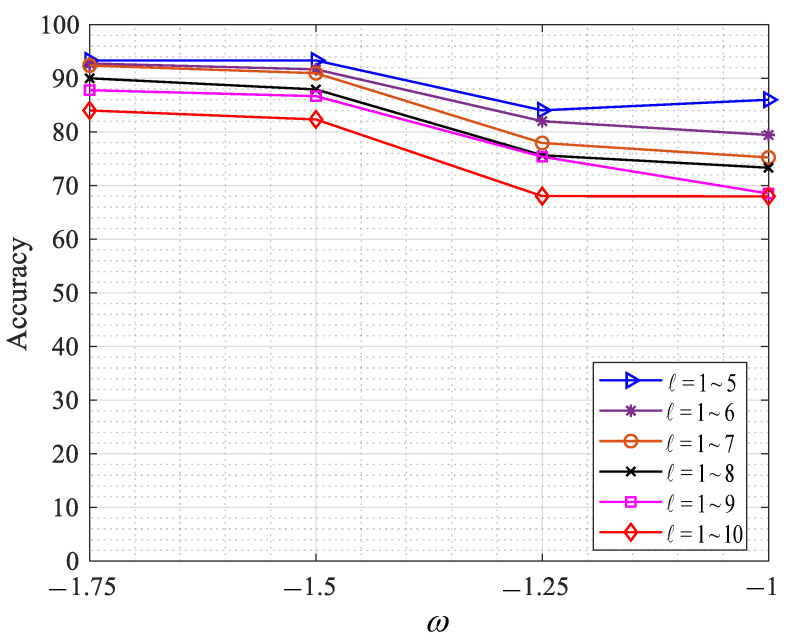
Recognition effect ω of OAM in different order modes.

**Figure 9:**
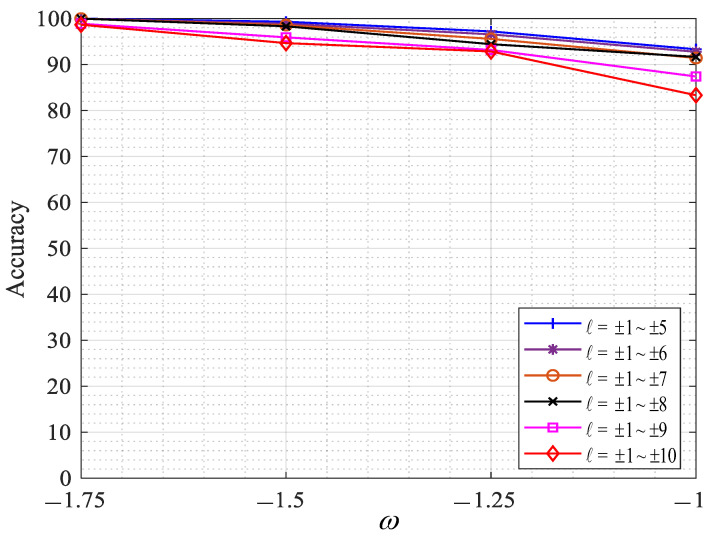
Dual-mode OAM recognition under different aspects ω.

**Figure 10:**
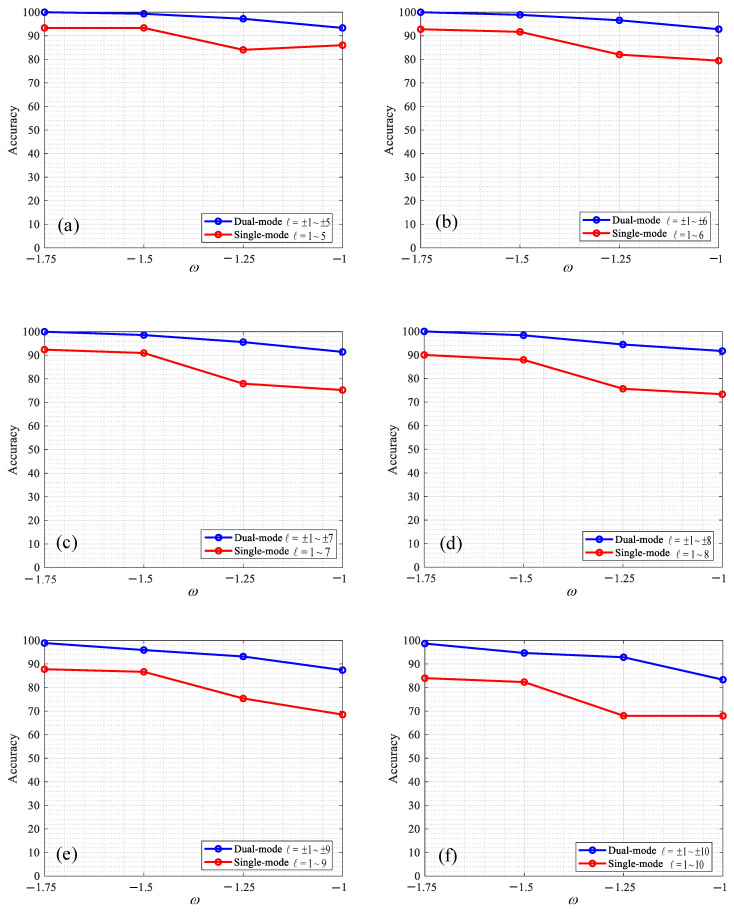
Comparison of dual-mode and single-mode OAM recognition results under different ω: (**a**) is the comparison chart of dual-mode (ℓ = ±1~±5) and single-mode (ℓ = 1~5); (**b**) is a comparison chart of dual mode (ℓ = ±1~±6) and single mode (ℓ = 1~6); (**c**) is a comparison chart of dual mode (ℓ = ±1~±7) and single mode (ℓ = 1~7); (**d**) is a comparison chart of dual mode (ℓ = ±1~±8) and single mode (ℓ = 1~8); (**e**) is a comparison chart of dual mode (ℓ = ±1~±9) and single mode (ℓ = 1~9); (**f**) is a comparison chart of dual mode (ℓ = ±1~±10) and single mode(ℓ = 1~10).

In this study, the dual-mode and single-mode OAM recognition methods based on CNN were studied, and the accuracy of OAM recognition changed with the variations. The parameters are set as follows: the wavelength was set to 532 nm, the transmission distance z was 100 m, the spacing of the phase screen was set to 10 m, and the phase screen size L was set to 0.04 m, the number of single-sided sampling points N of the phase screen was 1024, and the input image size was 128 × 128. The ratio of training set to test set in each group was 8:2, and the OAM modal recognition rate was obtained under the different ω values of turbulence intensity $C_{n}^{2}=1.0\times 10^{-13}K^{2}m^{-2/3}$. The experimental results are shown in Figure 8, Figure 9 and Figure 10.

According to Figure 8, when the intensity of ocean turbulence is $C_{n}^{2}=1.0\times 10^{-13}K^{2}m^{-2/3}$, the recognition rate of single-mode OAM (ℓ = 1~5, 1~6, 1~7, 1~8, 1~9, 1~10) is shown in different values ω, and in the case of ω = −1.75, single-mode OAM (ℓ = 1~5, 1~6, 1~7, 1~8, 1~9, 1~10) has higher recognition rates of 93.33%, 92.77%, 92.33%, 90%, 87.78%, and 84%, respectively, which can be observed. With the increase in the ratio of turbulence intensity ω caused by temperature salinity, the recognition accuracy of single-mode OAM gradually decreases, and with the increase in the number of recognized OAM patterns (ℓ = 1~5 to ℓ = 1~10), the recognition accuracy of the overall OAM shows a downward trend. Even under the conditions of strong turbulence, a good recognition effect can still be obtained, and under the disturbance of temperature and salinity of ω = −1.0, the OAM recognition rate of topological charge ℓ = 1~5 can reach 93.33%.

According to Figure 9, when the intensity of ocean turbulence is $C_{n}^{2}=1.0\times 10^{-13}K^{2}m^{-2/3}$, the recognition rate of dual-mode OAM (ℓ = ±1~±5, ±1~±6, ±1~±7, ±1~±8, ±1~±9, ±1~±10) under different values is ω = −1.75 and dual-mode OAM (ℓ = ±1~±5, ±1~±6, ±1~±7, ±1~±8, ±1~±9, ±1~±10) has higher recognition rates, which are 100%, 100%, 100%, 100%, 98.89%, and 98.67%, respectively. It can be observed from the figure that, with the increase in the ratio ω of turbulence intensity caused by temperature salinity, the recognition accuracy of dual-mode OAM gradually decreases, and with the increase in the number of recognized OAM patterns, the recognition accuracy of the overall OAM gradually decreases, but the overall dual-mode recognition effect is much better than the single-mode recognition effect.

Figure 10 shows the comparison of OAM recognition results between dual mode and single mode under different ω and the comparison of recognition rates between dual mode (ℓ = ±1~±5, ±1~±6, ±1~±7, ±1~±8, ±1~±9, ±1~±10) and single mode OAM (ℓ = 1~5, 1~6, 1~7, 1~8, 1~9, 1~10) under different ω values (ω = 1.75, −1.5, −1.25, −1.0). In Figure 10a, the recognition rates of dual-mode OAM (ℓ = ±1~±5) are 100%, 99.33%, 97.22%, and 93.33% and the rates of single-mode OAM (ℓ = 1~5) are 93.33%, 93.33%, 84.03%, and 86%, respectively. In Figure 10b, the recognition rates of dual-mode OAM (ℓ = ±1~±6) are 100%, 98.89%, 96.58%, and 92.78% and the recognition rates of single-mode OAM (ℓ = 1~6) are 92.77%, 91.67%, 82.00%, and 79.44%, respectively. In Figure 10c, the recognition rates of dual-mode OAM (ℓ = ±1~±7) are 100%, 98.57%, 95.59%, and 91.43% and the recognition rates of single-mode OAM (ℓ = 1~7) are 92.38%, 90.95%, 77.94%, and 75.24%, respectively. In Figure 10d, the recognition rates of dual-mode OAM (ℓ = ±1~±8) are 100%, 98.33%, 94.44%, and 91.67% and the recognition rates of single-mode OAM (ℓ = 1~8) are 90.00%, 87.92%, 75.65%, and 73.33%, respectively. In Figure 10e, the recognition rates of dual-mode OAM (ℓ = ±1~±9) are 98.89%, 95.92%, 93.19%, and 87.41% and the recognition rates of single-mode OAM (ℓ = 1~9) are 87.78%, 86.67%, 75.38%, and 68.52%, respectively. In Figure 10f, the recognition rates of dual-mode OAM (ℓ = ±1~±10) are 98.67%, 94.67%, 92.68%, and 83.33% and the recognition rates of single-mode OAM (ℓ = 1~10) are 84%, 82.33%, 68.03%, and 68%, respectively. It can be clearly seen from the figures that the recognition accuracy of dual-mode OAM is higher than that of single-mode OAM, and the anti-turbulence interference performance is better, because the phase diagram of dual-mode OAM not only has the features of a helical phase front but also of dual-mode superposition. Through experiments, it can be concluded that the more features to identify OAM, the better the effect for CNN.

As shown in Table 1 and Table 2, the single-mode OAM recognition rate at z=100 m in the ocean turbulence channel is characterized. Combined with Figure 10, according to the data shown in Table 1, the experimental results show that in the ocean turbulence channel, with the increase in ω value, the phase distortion of the *LG* beam is more serious, the phase helix feature is damaged, the OAM recognition rate decreases, and the larger the number of modes, the lower the recognition rate of OAM modes.

The results show that among the ocean turbulence channels dominated by temperature fluctuations, ocean turbulence has less influence on OAM recognition based on CNN. Conversely, among the ocean turbulence channels dominated by salinity fluctuations, ocean turbulence has a greater influence on CNN-based OAM recognition.

## 4. Conclusions

Section 4 summarizes the significance of this work and then considers areas of study related to those in this paper.

This paper examines the recognition of OAM using a CNN model, expounds the research status of CNN in OAM recognition, constructs a random phase screen model of ocean turbulence, takes the *LG* beam phase map through ocean turbulence as a dataset, extracts the spiral wavefront and phase singularity features of the phase map based on the CNN model, and simulates and analyzes the OAM recognition effect under different ω conditions. The results show that, under strong turbulence $C_{n}^{2}=1.0\times 10^{-13}K^{2}m^{-2/3}$, a good recognition effect can still be obtained, and the dual-mode OAM (ℓ = ±1~±10) recognition can reach 98.67%, even under a disturbance of temperature and salinity ω = −1.0, and the dual-mode OAM (ℓ = 1~10) recognition rate can reach 83.33%. Dual-mode OAM has higher recognition accuracy than single-mode OAM and has better anti-turbulence interference performance. The results can provide a reference for the study of optical communication technology of high-capacity OAM. In future studies, the influence of absorption, scattering, attenuation, and other factors can be further considered. It is believed that with continuous exploration and experimentation, the underwater information transmission rate will be greatly improved in the near future.

## Figures and Tables

**Table 1:** Recognition accuracy of dual-mode OAM.

ω	ℓ = 1~5	ℓ = 1~6	ℓ = 1~7	ℓ = 1~8	ℓ = 1~9	ℓ = 1~10
−1.75	100.00	100.00	100.00	100.00	0.9889	0.9867
−1.5	0.9933	0.9889	0.9857	0.9833	0.9592	0.9467
−1.25	0.9722	0.9658	0.9559	0.9444	0.9319	0.9286
−1.0	0.9333	0.9278	0.9143	0.9167	0.8741	0.8333

**Table 2:** Recognition accuracy of single-mode OAM.

ω	ℓ = 1~5	ℓ = 1~6	ℓ = 1~7	ℓ = 1~8	ℓ = 1~9	ℓ = 1~10
−1.75	0.9333	0.9278	0.9238	0.9000	0.8778	0.8400
−1.5	0.9333	0. 9167	0.9095	0.8792	0.8667	0.8233
−1.25	0.8403	0.8200	0.7794	0.7565	0.7538	0.6803
−1.0	0.8600	0.7944	0.7524	0.7333	0.6852	0.6800

## Data Availability

Some codes generated or used during this study are available from the corresponding author upon request.
